# The alarmin tandem: unraveling the complex effect of S100A8/A9 – from atherosclerosis to cardiac arrhythmias

**DOI:** 10.3389/fimmu.2025.1630410

**Published:** 2025-08-28

**Authors:** Dan-Alexandru Cozac, Vasile-Bogdan Halațiu, Alina Scridon

**Affiliations:** ^1^ Doctoral School of Medicine and Pharmacy, George Emil Palade University of Medicine, Pharmacy, Science, and Technology of Targu Mures, Targu Mures, Romania; ^2^ Physiology Department, George Emil Palade University of Medicine, Pharmacy, Science, and Technology of Targu Mures, Targu Mures, Romania; ^3^ First Cardiology Department, Emergency Institute for Cardiovascular Diseases and Transplantation of Targu Mures, Targu Mures, Romania; ^4^ Cardiology Department, Targu Mures Emergency Clinical County Hospital, Targu Mures, Romania; ^5^ Center for Advanced Medical and Pharmaceutical Research, George Emil Palade University of Medicine, Pharmacy, Science, and Technology of Targu Mures, Targu Mures, Romania

**Keywords:** arrhythmias, atherosclerosis, inflammation, S100A8/A9, heart failure

## Abstract

**Introduction:**

Inflammation plays a crucial role in the pathophysiology of cardiovascular diseases (CVDs), particularly in heart failure (HF), cardiac arrhythmias, and atherosclerotic cardiovascular disease (ASCVD). The calcium-binding proteins S100A8 and S100A9, primarily functioning as a heterodimer (S100A8/A9), have emerged as essential mediators in cardiovascular pathophysiology through the toll-like receptor 4 (TLR-4) and receptor for advanced glycation end-products (RAGE) signaling pathway. This review aims to comprehensively explore the role of S100A8/A9 in ASCVD, HF, and cardiac arrhythmogenesis, and to discuss its pathophysiological implications, clinical significance, and potential utility as a novel therapeutic target.

**Main text:**

In ASCVD, S100A8/A9 promotes endothelial dysfunction and facilitates monocyte recruitment and foam cell formation. The heterodimer amplifies vascular inflammation *via* TLR4 and RAGE signaling cascades, culminating in nuclear factor-kappa B activation and upregulation of proinflammatory cytokines that contribute to plaque instability. In HF patients, elevated S100A8/A9 levels correlate with disease severity and adverse outcomes through mechanisms involving cardiomyocyte death and pathological cardiac remodeling. Emerging evidence also implicates S100A8/A9 in cardiac arrhythmogenesis through electrical remodeling and pro-fibrotic effects. Despite significant advances in understanding the role of S100A8/A9 in cardiovascular pathology, significant knowledge deficiency remains. Further research is needed to elucidate cardiac-specific effects, temporal expression, and potential therapeutic applications.

**Conclusion:**

S100A8/A9 plays a critical dual role in cardiovascular inflammation and repair, emerging not only as a biomarker but also as a promising therapeutic target in ASCVD, HF, and cardiac arrhythmogenesis, with potential applications for anti-inflammatory intervention. However, further research is needed to elucidate the precise mechanisms linking S100A8/A9 and CVDs and to validate therapeutic interventions targeting this pathway.

## 1 Introduction

Cardiovascular diseases (CVDs) impose a substantial social and economic burden, standing as the leading cause of global mortality and morbidity (1, 2). Inflammation plays a crucial role in the pathophysiology of CVDs, particularly in heart failure (HF), cardiac arrhythmias, and atherosclerotic cardiovascular disease (ASCVD) (3–5). Recent data emphasizes the role of proinflammatory cytokines in the initiation, progression, and complications of these conditions (6, 7). Whether inflammation represents a risk factor or rather a risk modifier for CVDs remains unclear, and further research is needed to fully understand the complex impact of proinflammatory cytokines in CVDs and their potential as therapeutic targets. Recent clinical trials support the role of anti-inflammatory blockade in ASCVD, and the 2024 European Society of Cardiology guideline for the management of chronic coronary syndromes upgraded the class of recommendation for anti-inflammatory drugs (colchicine) to IIa class of recommendation (level of evidence A) (8–11). Several other clinical trials are ongoing, including in patients with acute coronary syndromes (ACS) (12, 13). However, the therapeutic anti-inflammatory arsenal remains limited, although preclinical studies constantly identify novel therapies that could guide cardiovascular treatments in a targeted and personalized manner.

Calprotectin, also known as S100A8/A9 is a heterodimeric complex of calcium-binding proteins that has recently emerged as a promising mediator of cardiac inflammation through the Toll-like receptor 4 (TLR-4) and receptor for advanced glycation end-products (RAGE) signaling pathway (14, 15). Research conducted over the last decade emphasizes its role as both a mechanistic mediator of cardiovascular pathology and a potential biomarker or even therapeutic target in various cardiac diseases (16, 17). However, its role as a biomarker for cardiovascular disease severity and therapeutic target via short-term blockade strategies to ameliorate inflammation represents a key area of ongoing investigation that warrants further exploration.

This review aims to comprehensively explore the role of S100A8/A9 in ASCVD and ischemic heart disease, HF, and cardiac arrhythmogenesis, and to discuss its pathophysiological implications, clinical significance, and potential utility as a therapeutic target to improve cardiovascular outcomes. An extensive literature search was conducted using PubMed, Web of Science, and Scopus databases. Search terms included “calcium-binding proteins,” “S100A8/A9”, “calprotectin,” combined with “cardiovascular disease,” “atherosclerosis,” “heart failure,” and “arrhythmia” using Boolean operators. Inclusion criteria were studies investigating calcium-binding proteins in cardiovascular pathology, and both experimental and clinical research.

### 1.1 Structure and function of S100A8/A9

Myeloid-related protein (MRP)-14, also known as S100A9 or calgranulin B, and MRP-8, S100A8 or calgranulin A, are members of the alarmin family and of the S100 family of calcium-modulated proteins, being expressed especially in cells of myeloid origins, such as the monocytes and neutrophils (14). Biochemically, S100A8 and S100A9 possess two calcium-binding domains and, in the absence of calcium ions, human S100A8/A9 remains in a heterodimer state (18). Experimental studies demonstrated that both humans and mice can assemble S100A8 and S100A9 to form the S100A8/A9 heterodimer, the predominant complex, which, due to its stability, is significantly more abundant than the respective homodimers (19, 20). Upon inflammatory stimuli, monocytes and neutrophils release both S100A8 and S100A9. The subsequently formed heterodimers can regulate the myeloid function through intracellular calcium signaling, facilitating cytoskeletal alterations and acting as a chemoattractant (21, 22). Released extracellularly, S100A8/A9 can interact with essential receptors like TLR-4 and RAGE, leading to nuclear factor-κB (NF-κB) activation and cytokine release (19, 21, 22). In addition, the S100A8/A9 complex is also involved in leukocyte recruitment, endothelial dysfunction, and oxidative stress, all of which contribute to CVD pathophysiology (23, 24). Therefore, elevated circulating S100A8/A9 levels have been linked to increased cardiovascular risk (such as heart failure incidence and progression) and disease severity (such us infarct size and functional impairment) (25, 26).

## 2 Role of S100A8/A9 in acute and chronic coronary syndromes

**Figure 1:**
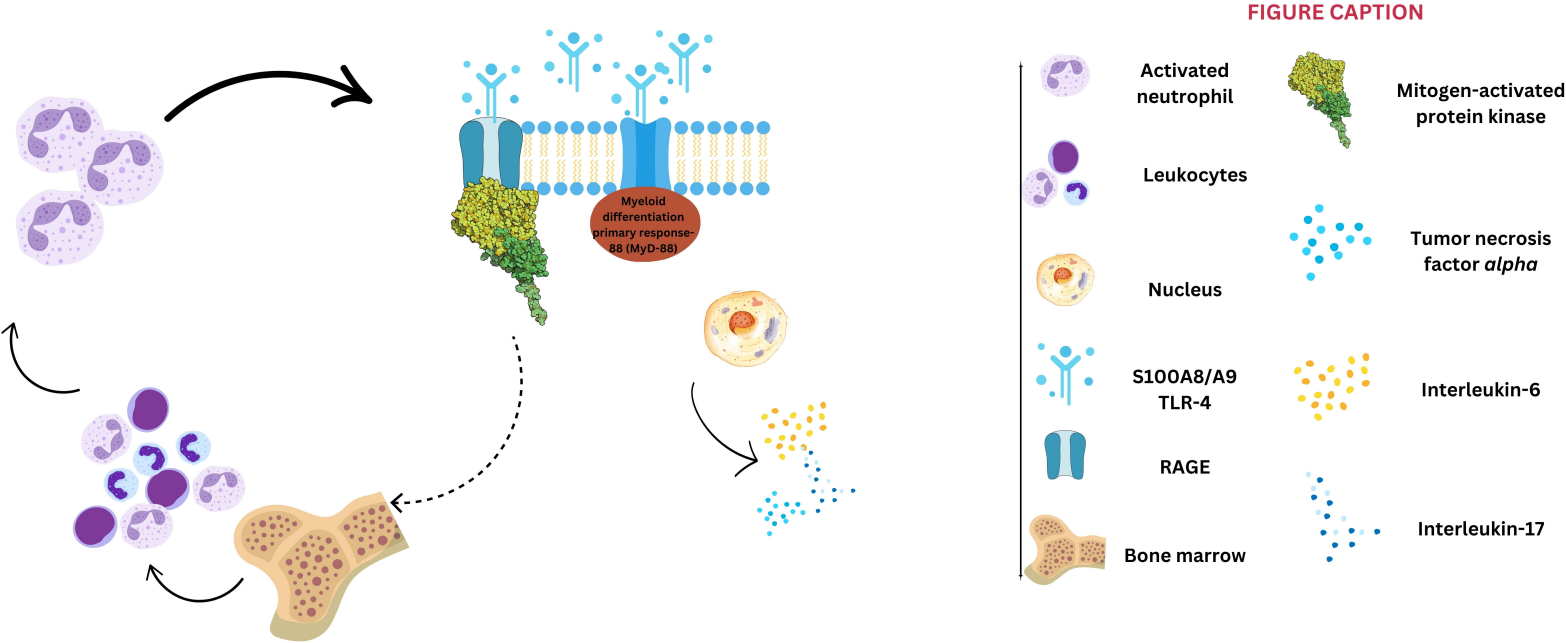
S100A8/A9 signaling pathways. S100A8/A9 activates two primary pathways: 1) binding to toll-like receptors 4 (TLR-4) triggers the myeloid differentiation primary response-88 dependent signaling cascade, culminating with nuclear factor κ *beta* activation and subsequent upregulation of interleukin-6, -17, and tumor necrosis factor-alpha; and 2) interaction with receptor for advanced glycation end-products (RAGE) stimulates the mitogen-activated protein kinase pathway, further contributing to leukocyte proliferation within bone marrow, and a positive feedback for S100A8/A9 synthesis.

The inflammatory theory of atherosclerosis and the involvement of different immune cells in atherosclerosis pathophysiology has been confirmed in experimental and clinical studies (27, 28). The first immune gatekeepers are the M1 subtype of macrophages, which invade the lipid streaks, the earliest atherosclerotic lesions (29). These cells engulf the oxidated low-density lipoproteins, transform into foam cells, and promote further development of atherosclerotic vascular plaques by releasing proinflammatory cytokines and contributing to acute plaque events (27–29). The interaction of S100A8/A9 with TLR-4 activates the myeloid differentiation primary response-88 (MyD-88)-dependent signaling cascade, culminating in NF-κB activation and upregulation of proinflammatory cytokines such as the tumor necrosis factor-alpha (TNF-α), interleukin (IL)-6, and IL-17 (30, 31). Additionally, S100A8/A9 binds to RAGE, stimulating the mitogen-activated protein kinase (MAPK) intracellular pathway, which enhances leukocyte proliferation within the bone marrow and drives further S100A8/A9 synthesis (**Figure 1**), perpetuating the inflammatory cascade (31). The key role of the RAGE pathway in the atherosclerotic process was shown in murine hypercholesterolemic models, in which the inhibition of this pathway was associated with reduced atherosclerotic plaque progression (32, 33). In addition, S100A8/A9 plays a crucial role in neutrophil extracellular trap (NET) formation and activation, inducing neutrophil activation, adhesion, and chemotaxis (34). Following NET formation, the released components, including histones, proteases, and coagulation factors – subsequently contribute to the development and progression of atherosclerosis, driving both plaque formation and arterial thrombosis (34). S100A8/A9 is upregulated in response to myocardial ischemia, promoting endothelial activation, platelet aggregation, and thrombus formation. Studies indicate that patients with acute ACS have significantly higher plasma S100A8/A9 levels, which correlate with infarct size and adverse outcomes (35, 36). Platelets play a crucial role in atherogenesis and thrombosis-mediated myocardial ischemia (37). An experimental study provided evidence that neutrophil-derived S100A8/A9 triggers thrombocytosis in diabetic murine models through RAGE-mediated signaling pathways, which induce IL-6 production and subsequent stimulation of hepatic thrombopoietin synthesis. This preclinical approach underscores the intricate relationship between S100A8/A9 and the pathogenesis of atherosclerosis (38).

**Figure 2:**
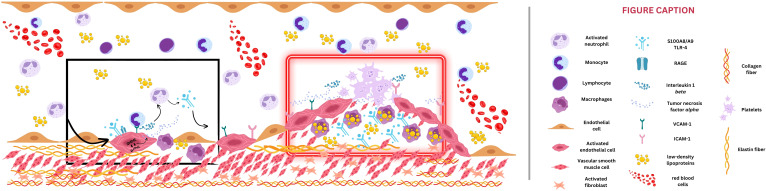
Potential mechanism of S100A8/A9-induced atherosclerosis. The black box depicts the early phase of atherosclerosis. S100A8/A9 induces endothelial activation and secondary adhesion molecule expression (vascular cell adhesion protein 1 [VCAM-1] and intercellular adhesion molecule 1 [ICAM-1]), which will enable monocytes to adhere to the vessel wall, transform into macrophages, and engulf particles of low-density lipoproteins. Through S100A8/A9, intracellular signaling such as toll-like receptor-4 (TLR-4) and receptor for advanced glycation end-products (RAGE) activation will be responsible for reactive oxygen species (ROS) release and will create a continuous loop of vessel inflammation. The red box emphasizes an advanced stage of atherosclerotic plaque formation, with narrowed vessel lumen. S100A8/A9 amplifies the inflammatory status by interleukin-6 and tumor necrosis factor *alpha* release, smooth muscle cell migration toward the plaque, and extracellular matrix degradation and lysis of the fibrous cap, culminating with plaque rupture.

By interacting with vascular cells, S100A8/A9 also contributes to atherosclerotic plaque progression. Promoting the expression of adhesion molecules and facilitating the recruitment of immune system cells, S100A8/A9 exacerbates endothelial dysfunction (39). Activation of the mammalian target of rapamycin (mTOR)-2 system determines the phosphorylation of protein kinase B (Akt), leading to increased protein synthesis, cytoskeletal reorganization through activation of certain hydrolase enzymes, and increased expression of endothelial cell cycle regulators (e.g., cyclin D1) (40). Also, the hypoxia-inducible factor 1 (HIF-1) seems to play a crucial role in the pro-angiogenic effect of S100A8/A9, regulating vascular growth factor (VEGF) expression and angiogenesis, but also in the activation of matrix metalloproteinases and extracellular matrix organization (41). Therefore, by promoting cell growth and angiogenesis via RAGE signaling and activation of the mTOR-2, S100A8/A9 promotes intimal hyperplasia, an essential step of atherosclerosis (**Figures 1**, **2**). This process is characterized by abnormal vascular smooth muscle cells (VSMCs) migration from the vascular media to the intima and their consecutive transformation from a contractile to a synthetic state (42). Finally, S100A8/A9-induced modulation of VSMCs is associated with instability of the fibrous cap of the vascular plaque and potential plaque rupture. Moreover, S100A8/A9 contributes to altered blood flow through vasoconstriction via endothelial dysfunction and enhanced platelet-leukocyte aggregates. Thus, S100A8/A9 modulates all three components of Virchow’s triad, ultimately contributing to atherothrombosis and subsequent ACS (**Figure 2**).

The recent findings and implications of S100A8/A9 in the atherosclerotic process have spurred studies focused on investigating the role of S100A8/A9 in ACS patients. The involvement of various immune cell populations in both the initiation and progression of atherosclerosis encompasses highly complex and interconnected mechanisms that act at multiple stages of disease development. S100A8/A9 drives extensive inflammation during ACS via the TLR-4 and RAGE pathways (**Figure 1**), amplifying neutrophil and monocyte activation, cytokine secretion, and enhanced granulopoiesis (31, 35). All these can contribute to cardiomyocyte necrosis, mitochondrial dysfunction, and left ventricular systolic dysfunction post-ACS (43).

Healy et al. demonstrated in a platelet transcriptome model that S100A9 is a strong discriminator of acute ST-segment elevation myocardial infarction (STEMI) patients and higher concentrations of S100A8/A9 were associated with worse cardiovascular outcomes (44). These results were also confirmed by Sakuma et al., who highlighted the importance of S100A8/A9 as a potential biomarker in ACS. In their study, S100A8/A9 levels measured in coronary artery blood distal to the culprit lesion were higher in patients with thrombus compared to those without thrombus (45). These findings support the promising role of S100A8/A9 as a potential treatment target for ACS inflammation modulation. However, that study has several limitations, particularly the fact that the higher plasma concentration of S100A8/A9 observed in patients with aspirated thrombus may have been due to sampling errors, given that thrombus aspiration is not always successful.

Another mechanistic study characterized and described the platelet proteome in ACS, showing that releasing the S100A8/A9 heterodimer was associated with decreased ex vivo platelet activation. The same working group demonstrated for the first time that, in STEMI patients, the plasma levels of S100A8/A9 were double compared to patients with stable ASCVD, and that platelet micro ribonucleic acid (mRNA) levels for S100A8 and S100A9 were increased at the time of STEMI compared to the levels measured on the third day post-event (45). However, further research is necessary to determine whether the platelet reactivity induced by S100A8/A9 can be used to phenotype patients with high-risk features of plaque rupture and complications.

Sreejit and collaborators demonstrated on a mouse model of myocardial infarction that neutrophil-derived S100A8/A9 amplifies acute inflammation, by stimulating myeloid progenitor cells in the bone marrow, which lead to enhanced granulopoiesis (46). Since S100A8/A9 seems necessary for ACS-induced granulopoiesis, they highlighted the potential beneficial effects of interrupting the S100A8/A9-granulopoiesis axis in reducing the myocardial scar. Subsequently, in their experimental study, the infarct size of mice with disrupted S100A8/A9-granulopoiesis axis presented a significantly smaller scar, higher ventricular systolic performance, and better cardiac outcomes (46) However, these results are limited by the study design, given that the infarct model was obtained using ligature of the left anterior descending artery and did not involve atherosclerotic plaque rupture, the most prevalent pathophysiology in patients presenting with ACS.

The ischemia/reperfusion injury represents another promising S100A8/A9 target for reducing myocardial damage after coronary revascularization. Even though animal models have indicated promising molecular interventions to reduce the burden of myocardial ischemia after ischemia/reperfusion injury, data on clinically effective strategies are scarce. Li et al. studied the role of S100A8/A9 in myocardial necrosis secondary to reperfusion injury. The authors used complementary experimental approaches to establish robust evidence for S100A8/A9’s role in ischemia/reperfusion injury (47). In that study, S100A8/A9 enhanced ischemia/reperfusion injury and myocyte necrosis through impaired activity of the mitochondrial complex I (47). Mitochondrial permeability transition pore opening (mPTP), with subsequent alterations in mitochondrial potential, led to increased reactive oxygen species (ROS) and impaired adenosine triphosphate synthesis (48). By inducing cytochrome c releasing and activation of caspase-enzymes, S100A8/A9 is also involved in apoptosis pathways (49, 50). In their study, Li et al. emphasized that S100A8/A9 overexpression was associated with increased severity of ischemia/reperfusion injury, while genetic ablation and pharmacological inhibition of S100A9 conferred myocyte protection (47). These molecular relationships were characterized by a complex interplay between S100A8 and S100A9, because S100A9 knockout mice exhibited concurrent depletion of S100A8, whereas S100A8 stability critically depends on the S100A9 effect (47). Therefore, they have highlighted the concept of receptor-mediated amplification, where S100A9’s association with RAGE initiates positive feedback, enhancing both S100A8 and S100A9 production and creating a self-perpetuating inflammatory cascade. Neutralizing antibodies against S100A9 administered in wild-type mice were associated with reduced myocardial scar and subsequent cardiac fibrosis (47). Therefore, new insights into the complexity of the effects of S100A8/A9 in ACS pathophysiology have been brought, with important clinical implications such as the development of specific S100A8/A9 inhibitors for ischemia/reperfusion injury. However, incomplete understanding of all downstream effectors, limited exploration of potential compensatory mechanisms, and limited investigation of other cardiac cells contribute to a need for further research. This approach should focus on elucidating the signaling complexity and cell-type specificity of S100A8/A9 pathways in ischemia/reperfusion injury.

Recently, an experimental approach was used to test whether pharmacological inhibition of S100A8/A9 could attenuate the inflammatory cascade and enhance myocardial performance in the post-infarction recovery phase (36, 51, 52). Marinkovic et al. demonstrated that three-day selective inhibition of S100A8/A9 using ABR-238901 had beneficial effects on cardiac function after myocardial infarction and stimulated reparative phases in the injured myocardium (51). This mechanistic investigation delivered new evidence into the therapeutical modulation of the S100A8/A9 pathway in ACS. A similar study by Chalise et al. demonstrated in a murine myocardial infarction model that S100A8/A9 is rapidly released after the onset of acute myocardial ischemia and correlates with infarct wall thinning (53). Moreover, the authors emphasized the role of S100A9 in stimulating neutrophils and macrophage influx rather than in directly triggering neutrophil degranulation. S100A9 acts as a direct modulator of infarct wall thinning by influencing extracellular matrix remodeling (53). Therefore, targeting this protein may help promote infarct healing in the myocardial border zone. However, Schiopu et al. demonstrated that only short-term therapeutic modulation of S100A9 is beneficial, whereas S100 A9 extended blockage can lead to progressive deterioration of cardiac systolic function and subsequent ventricular dilatation (24, 36). The temporal analysis of S100A9 effects describes a critical intervention period corresponding to the acute inflammatory response in ACS. This defined therapeutic window suggests that targeted therapeutical modulation of S100A9 during the early inflammatory response represents an optimal timing for immunomodulatory intervention in ACS. The evidence strongly supports that temporal considerations are critical for S100A8/A9 blockade efficacy and safety. Short-term intervention (3–14 days) initiated after the acute defense phase appears optimal, preserving beneficial functions while preventing pathological consequences. However, further studies are needed to validate these results in larger preclinical models for translational robustness. Furthermore, appropriate clinical translation requires precise timing, appropriate patient selection, and recognition that S100A8/A9 serves essential physiological functions. Schiopu et al.’s investigation provided a novel perspective on the previous unsuccessful studies to develop new anti-inflammatory drugs for ACS (24). Their study emphasized that the failure to translate pathophysiological findings into effective clinical treatments might be attributed to unsuitable treatment duration. This represents a paradigm shift in approaching anti-inflammatory therapy in ACS, by also focusing on maintaining the reparative process essential for adequate myocardial healing, suggesting that modulation, rather than complete suppression of inflammatory response, is the key for optimal cardiac benefits. Therefore, the biomarker utility of S100A8/A9 is well-supported to guide risk stratification and outcome prediction in ACS patients and existing data highlights the concept of temporally targeted immune modulation.

Recent studies also highlighted the association of S100A8/A9 with the occurrence of in-stent thrombosis (54). Wang et al. discovered an abrupt increase in S100A8/A9 levels in patients with late stent thrombosis, compared with the index percutaneous coronary intervention procedure for ACS. However, in multivariate Cox regression analysis, even though S100A8/A9 plasma level was a predictor of intrastent thrombosis, the hazard ratio was 1.001, which emphasizes a rather neutral effect of S100A8/A9 in predicting late stent thrombosis. Also, S100A8/A9 was independently associated with recurrent myocardial infarction or cardiovascular death in a subgroup analysis of patients enrolled in the Pravastatin or Atorvastatin Evaluation and Infection Therapy: Thrombolysis in Myocardial Infarction (PROVE-IT TIMI 22 trial), suggesting the multifaced role of this heterodimer (55).

The studies mentioned above highlight the complex involvement of S100A8/A9, which seems to play a crucial role in ACS by inducing subsequent inflammation and worsening myocardial injury, while short-term therapeutic modulation of this heterodimer seems to reduce inflammatory damage and improve cardiovascular outcomes, at least in experimental models. While nanoparticles carrying small interfering RNA molecules that target S100A8/A9 showed promise in reducing myocardial injury and local inflammation in experimental models, clinical trials are still necessary to prove their effectiveness. **Table 1** provides the most relevant studies investigating the role of S100A8/A9 in ASCVD pathogenesis.

**Table 1:** Summary of key studies on S100A8/A9 contribution to atherosclerosis, heart failure, and cardiac arrhythmias.

Authors/year	Title	Study design	Population/Experimental model	Key findings
S100A8/A9’s role in atherosclerosis cardiovascular disease				
McCormick et al., 2005 (56)	S100A8 and S100A9 in Human Arterial Wall	Experimental (*in vitro* and human tissue analysis)	39 patients undergoing endarterectomy and 14 undergoing aortic reconstruction	S100A8/A9 present in atherosclerotic plaques, associated with calcification. influences redox processes
Miyamoto et al., 2008 (57)	Increased Serum Levels and Expression of S100A8/A9 Complex in Infiltrated Neutrophils in Atherosclerotic Plaque of Unstable Angina	Observational, clinical study	39 patients with stable angina, 53 patients with unstable angina	Increased serum S100A8/A9 levels in unstable angina, associated with neutrophil infiltration in plaques
Nagareddy et al., 2013 (58)	Hyperglycemia Promotes Myelopoiesis and Impairs the Resolution of Atherosclerosis.	Dual: animal model study. observational clinical study	Diabetic mice; patients with type 1 diabetes	S100A8/A9 production enhanced by hyperglycemia, promotes myelopoiesis and atherogenesis
New et al. 2013 (59)	Macrophage-Derived Matrix Vesicles: An Alternative Novel Mechanism for Microcalcification in Atherosclerotic Plaques	Dual: *in vitro* experimental study; animal model study. observational clinical study	136 patients with carotid plaques; S100A9−/− mice	Macrophage-derived S100A9 contributes to microcalcification in atherosclerotic plaques
Geczy et al. 2014 (60)	Calgranulins May Contribute Vascular Protection in Atherogenesis	Experimental model	Genetically modified mice S100A9 knockout (Low-Density Lipoprotein Receptor knockout, Apolipoprotein E-deficient	S100A8/A9 has pro- and antiinflammatory effects, influences leukocyte recruitment and oxidative stress
Oesterle et al., 2015 (61)	S100A12 and the 100/Calgranulins: Emerging Biomarkers for Atherosclerosis and Possibly Therapeutic Targets.	Dual: Animal model. observational study	Transgenic mice (S100A12, ApoE-/-), S100A9 or RAGE knockout mice	S100A8/A9-RAGE interaction mediates vascular inflammation
Chen et al. 2018 (62)	Arterial Thrombosis Is Accompanied by Elevated Mitogen-Activated Protein Kinase (MAPK) and Cyclooxygenase-2 (COX-2) Expression via Toll-Like Receptor 4 (TLR-4) Activation by S100A8/A9.	Dual: clinical study; *in* *vitro* experimental study	303 human participants (110 acute coronary syndrome, 110 coronary heart disease, 83 controls); Sprague- Dawley ratsș Human Aortic Endothelial Cells (HAECs)	S100A8/A9 upregulated after vessel injury, activates TLR4-MAPKCOX2 signaling, correlates with thrombosis
Marinkovic et al., 2019 (63)	Inhibition of pro-inflammatory myeloid cell responses by short-term S100A9 blockade improves cardiac function after myocardial	Experimental animal study with human observational elements	524 patients with acute coronary syndrome; wild-type C57BL/6 mice with myocardial infarction induced by permanent coronary artery ligation, treatment with the S100A9 blocker	In mouse models of MI, short-term S100A9 inhibition reduces inflammation and enhances heart function.
Marinkovic et al., 2019 (64)	S100A9 Links Inflammation and Repair in Myocardial Infarction	Experimental animal study with human observational elements	524 patients with confirmed acute coronary syndrome; Wild type C57BL/6 mice with permanent coronary artery ligation;	S100A9 plays a key function in MI and is a dual promoter of inflammation and repair.
Mares et al. 2024 (24)	Short-term S100A8/A9 Blockade Promotes Cardiac Neovascularization after Myocardial Infarction	Male and female wild-type (C57BL/6) mice	Ligaturation-induced myocardial infarction in male and female wild-type mice MI	Blocking S100A8/A9 is a crucial factor in promoting cardiac recovery following myocardial infarction.
S100A8/A9’s role in heart failure				
Ma et al. 2012 (26)	S100A8/A9 Complex as a New Biomarker in Prediction of Mortality in Elderly Patients with Severe Heart Failure	Human observational study (cohort)	108 participants: 54 congestive heart failure patients, 31 hypertensive patients without congestive heart failure, and 23 control subjects	S100A8/A9 identified as an independent predictor of mortality in elderly population with severe heart failure
Li et al. 2019 (47)	S100a8/A9 Signaling Causes Mitochondrial Dysfunction and Cardiomyocyte Death in Response to Ischemic/Reperfusion Injury	Experimental animal study with clinical observational elements	Mouse model of myocardial ischemia-reperfusion injury; patients with acute myocardial infarction	S100A8/A9 is an early mediator in myocardial ischemia-reperfusion injury
Frangogiannis et al. 2019 (23)	S100A8/A9 as a Therapeutic Target in Myocardial Infarction: Cellular Mechanisms, Molecular Interactions, and Translational Challenges	Dual: Experimental animal study + clinical observational elements	Mouse models of reperfused and non-reperfused myocardial infarction; patients with acute coronary syndromes	S100A8/A9 inhibition is associated with reduced inflammation and improved cardiac function post-myocardial infarction
Vallejo et al. *2023* (65)	The effects of S100A8/A9 blockade in experimental Myocardial Infarction detected by CITE-seq RNA sequencing	Experimental model	Murine model of myocardial infarction	S100A8/A9 Inhibition reduce inflammation and improves cardiac function post-myocardial infarction
Wu et al. 2023 (66)	S100a8/A9 Contributes to Sepsis- Induced Cardiomyopathy by Activating ERK1/2-Drp1-Mediated Mitochondrial Fission and Respiratory Dysfunction	Experimental model	S100A9 knockout and wild-type mice with sepsis-induced cardiomyopathy	S100A9-induced mitochondrial dysfunction plays a crucial role in sepsis-related cardiomyopathy
Voss et al. 2024 (67)	Role of the Alarmin S100A9 in the Pathogenesis of Heart Failure with Preserved Ejection Fraction.	Dual: experimental model; clinical observational study	Wild-type and S100A9 knockout mice; human endomyocardial biopsy samples	S100A8/9 contributes to inflammation in heart failure with preserved ejection fraction; knockout mice demonstrated less cardiac dysfunction
Ma et al. 2024 (26)	S100A8/A9 as a Prognostic Biomarker with Causal Effects for Post-Acute Myocardial Infarction Heart Failure	Clinical observational study with Mendelian randomization	Discovery cohort: 1,062 patients. Validation cohort: 1,043 patients; all with acute myocardial infarction	Increased levels of S100A8/A9 are associated with an increased risk of post-myocardial infarction heart failure
S100A8/A9’s role in cardiac arrhythmogenesis				
Flevari et al. 2019 (68)	P6596S100A8/A9 and sRAGE Peripheral Blood Levels in Patients with Heart Failure and an Implanted Cardioverter/Defibrillator: Relation with Sustained, Fast Ventricular Arrhythmias	Human observational study (cohort	60 patients with clinically stable heart failure and implanted implantable cardioverter defibrillators (ICDs)	Reduced S100A8/A9 Levels associated with higher risk of ventricular arrhythmias
Jakobsson et al., 2023 (69)	Therapeutic S100A8/A9 blockade inhibits myocardial and systemic inflammation and mitigates sepsis-induced myocardial dysfunction	Dual: experimental model; clinical prospective observational study	62 sepsis patients; female C57Bl/6NrJ mice	Blockade of S100A8/A9 with ABR−238901 has potent anti-inflammatory effects – possibly reducing arrhythmic burden
Boyd et al. 2008 (70)	S100A8 and S100A9 Mediate Endotoxin-Induced Cardiomyocyte Dysfunction via the Receptor for Advanced Glycation End Products	Experimental model	adult male mice injected IP with 40 mg/kg Escherichia coli–derived lipopolysaccharide	cardiomyocytes exposed to LPS express S100A8 and S100A9, leading to a RAGE-mediated decrease in cardiomyocyte contractility
Zhang et al. 2015 (71)	Necrotic Myocardial Cells Release Damage‐Associated Molecular Patterns That Provoke Fibroblast Activation *In Vitro* and Trigger Myocardial Inflammation and Fibrosis *In Vivo*	Experimental *in vivo* and *in vitro*	freshly excised mice hearts with induce myocardial necrosis and adult mice	Necrotic myocardial cells lead to fibroblast activation *in vitro* and myocardial inflammation and fibrosis *in vivo via* damage‐associated molecular patterns

## 3 Role of S100A8/9 in heart failure

Recent advances have significantly expanded our understanding regarding the complex relationship between inflammation and HF, highlighting the potential role of inflammation as a potential therapeutic target for new immunomodulatory strategies, especially in patients with HF with preserved ejection fraction (HFpEF) (72). Novel insights into inflammasome-dependent mechanisms and NET formation have revealed previously hidden pathways in cardiac dysfunction and, nowadays, chronic inflammation is recognized as a hallmark of HF (72). Accordingly, discovering new molecular modulators, such as specific cytokine networks and extracellular vesicles, seems to represent the next breakthrough in HF therapy.

**Figure 3:**
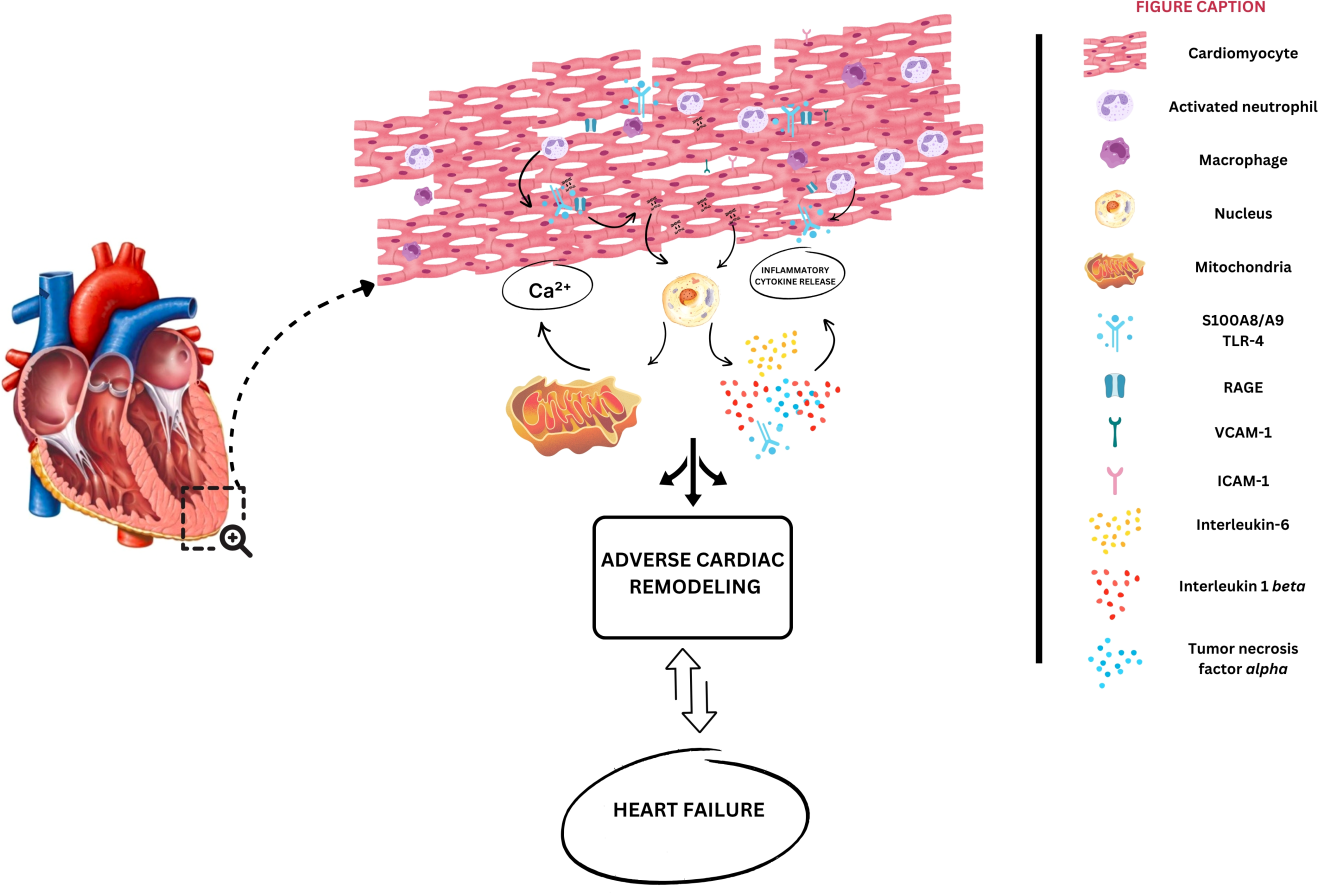
Potential mechanisms of S100A8/A9 contribution to heart failure development. Initial cardiac injury leads to neutrophil infiltration into cardiomyocytes, which will be responsible for S100A8/A9 release into the extracellular space. S100A8/A9 binds to the receptor for advanced glycation end-products (RAGE), activating toll-like receptor-4 (TLR-4), converging to nuclear factor kappa b activation (NF-kB). Consequently, interleukins 1 *beta*, and 6 are produced, in parallel with mitochondrial dysfunction and adenosine triphosphate (ATP) depletion. Sustained inflammation leads to fibrosis, cardiac hypertrophy, and cardiomyocyte dysfunction.

Mechanistically, S100A8/A9 plays a crucial role in ischemic HF pathogenesis through multiple mechanisms (**Figure 3**). After cardiac injury and subsequent neutrophil infiltration, S100A8/A9 initiates an inflammatory cascade through RAGE and TLR-4 pathways (**Figure 1**), resulting in oxidative stress mediated by NF-κB (15, 22, 30). Moreover, via induction of mitochondrial dysfunction by mPTP opening and intracellular calcium disturbances, S100A8/A9 is directly related to cardiomyocyte apoptosis (48). As previously highlighted, this heterodimer complex enhances the recruitment of inflammatory cells, participating in a continuous, self-perpetuating loop of inflammation (48). Furthermore, it contributes to adverse cardiac remodeling by activating fibroblasts and modulating extracellular matrix metabolism. Mediating the inflammation that exacerbates myocardial fibrosis and consequent ventricular dysfunction, S100A8/A9 shows promise as both a prognostic biomarker and a therapeutic modulation target, as supported by experimental and early clinical studies, particularly during the acute phase of inflammation.

Given the central role of prolonged inflammation in the progression of ASCVD, insights into ischemic HF have dominated the last decade of S100A8/A9 research in the HF syndrome, leading to a large body of evidence compared with other etiologies. **Table 1** provides the most important studies investigating the role of S100A8/A9 in HF pathogenesis and progression.

Pioneering research into mechanistic insights on S100A8/A9 contribution to the progression of ischemic HF was performed on murine myocardial infarction models. Volz et al. demonstrated the implication of the RAGE signaling pathway as the primary effect of S100A8/A9 in cardiomyocytes and its role in inducing fibrosis (73). By promoting NF-κB signaling pathways, S100A8/A9 leads to increased production of proinflammatory cytokines, increased oxidative stress, and a subsequent loop of maladaptive cardiac remodeling. Furthermore, the authors demonstrated that genetic ablation of S100A9 or RAGE pathway blockade was associated with cardioprotective effects, reduced fibrosis, and attenuated inflammatory response (70, 73). Pharmacological inhibition of the S100A8/A9 axis provided identical benefits, suggesting therapeutic modulation potential (74).

A recent study evaluated S100A8/A9 levels in patients with acute myocardial infarction and their relationship with *de novo* HF during 4.2 years of follow-up (26). The findings revealed S100A8/A9 as an independent predictor of subsequent HF, even after adjusting for traditional risk factors, such as infarct size, classic inflammatory biomarkers, and systolic ventricular performance, highlighting its potential value as a prognostic tool for patients with HF (26).

Similarly, Li-Ping Ma et al. examined S100A8/A9 levels in an elderly population with advanced HF (NYHA classes III-IV, both systolic and diastolic HF) and explored their role in predicting mortality (75). First, they demonstrated that elevated S100A8/A9 levels positively correlated with traditional inflammatory cytokines such as IL-6 and TNF-α. Second, in Cox regression, S100A8/A9 remained an independent predictor for one-year mortality, together with IL-6, suggesting the importance of a combined score to ensure a closer follow-up of these patients. A notable strength of their investigation was its focus on cardiac inflammation, specifically within an elderly population suffering from advanced HF, demonstrating that S100A8/A9 provides valuable additional prognostic insights beyond classical inflammatory biomarkers.

Preclinical models suggest that S100A8/A9 inhibition may attenuate adverse remodeling and improve cardiac function (23, 46, 65, 74). The role of targeted, short-term S100A9 inhibition on left ventricular systolic performance in an experimental model of myocardial infarction revealed that three days of inhibition of S100A9 during the acute inflammatory phase significantly modulates cardiac proteome changes in ways that favor myocardial recovery and repair processes. By modulating anti-apoptotic proteins involved in p53 signaling pathways and reducing the expression of molecules involved in leukocyte recruitment, S100A8/A9 blockade offers cardioprotective effects (76).

The implications of S100A8/A9 and soluble RAGE signaling pathways were also evaluated in patients with HF and subsequent malignant ventricular arrhythmias. Interestingly, the study of Flevari et al. revealed that S100A8/A9 levels were lower in patients with HF, implantable cardioverter defibrillator, and sustained ventricular arrhythmias during a four-year follow-up period, suggesting the potential role of S100A8/A9 as a prognostic biomarker of sudden cardiac death in this population (68). Nonetheless, these findings must be considered within all observational study limitations, together with a reduced sample size and limited rate of sustained ventricular arrhythmias.

Due to an incomplete understanding of the pathogenic mechanisms and subsequent ineffective therapies, HFpEF continues to be associated with high morbidity and mortality (77, 78). Accumulating evidence highlights that inflammation plays a central role in HFpEF, emphasizing that the burden of comorbidities induces a systemic proinflammatory state, contributing to coronary endothelial inflammation, myocardial stiffness, and consecutive myocardial fibrosis (79–82). Voss et al. demonstrated in a murine model of HF that the adipose tissue is an important extracardiac source of S100A9, which increases mRNA expression of chemokine-C ligand 2 (CCL2) and 7 (CCL7) in cardiac fibroblasts, potentially identifying a novel therapeutic target for HFpEF (67).

Inflammation-mediated HF is also closely associated with myocardial hypertrophy, particularly in patients with HFpEF, and this contribution could also stand as a therapeutic target. In mice with angiotensin II infusion, administration of an S100A9 antibody demonstrated protective effects against ventricular hypertrophy by suppressing infiltration with cluster of differentiation (CD) 45 leukocytes and CD11b monocytes and blocked NF-κB-dependent proinflammatory expression (25, 83). Complementary, Sun et al. demonstrated in an experimental model of cardiac fibrosis that S100A8/A9 blockade was associated with decreased levels of NF-κB p65 and reduced cardiac fibrosis (84).

Evolving evidence suggests that S100A8/A9 adds diagnostic value in all HF subtypes when combined with N-terminal pro brain natriuretic peptide (NTproBNP), but high plasma levels of this heterodimer are also elevated in myocarditis and several other inflammatory conditions (14). While NTproBNP level reveals hemodynamic status and troponin levels indicate myocardial injury, S100A8/A9 defines the inflammatory axis, offering an additional risk evaluation tool. The role of S100A8/A9 in patients with sepsis-induced cardiomyopathy and myocarditis was also studied. Muller et al. analyzed serum S100A8/A9 levels across patients with recent-onset myocarditis, and their research supports the role of this biomarker as a potential diagnostic tool with high sensitivity and specificity (85). Additionally, S100A8/A9 was shown to play a key role in Coxsackie virus B3-induced myocarditis in both experimental and human models, and it was recognized as a tool to track disease progression (86).

Sepsis triggers a systemic inflammatory response, which has the potential to trigger cardiomyocyte necrosis and impair systolic performance. S100A8/A9 seems to play a crucial role in sepsis-induced cardiomyopathy, and Wu et al. demonstrated in a murine model of sepsis that S100A8/A9 activates extracellular signal-regulated kinases 1/2 and dynamin-related protein 1 (ERK1/2-DRP1) signaling pathways, responsible for mitochondrial dysfunction. Also, the authors highlighted the beneficial effects of S100A9 inhibition on systolic function and mortality in their model of sepsis-induced cardiomyopathy (66).

The translation of experimental findings into effective clinical intervention remains challenging, but promising clinical findings support inflammation-targeted HF therapy. Recently, the Canakinumab Antiinflammatory Thrombosis Outcome Study trial has demonstrated promising results. Administration of canakinumab, a monoclonal antibody targeting IL-1β, was associated with a dose-dependent reduction in hospitalization and mortality in patients with HF (11). Even though S100A8/A9 seems to be a promising diagnostic and prognostic biomarker in HF, a gap in clinical translation exists. To date, no clinical trials have investigated targeted inhibition of S100A8/A9 in cardiovascular diseases, underscoring the need to assess the safety and efficacy of S100A8/A9 inhibitors in phase 2 trials. **Table 1** provides the most relevant studies investigating the role of S100A8/A9 in HF pathogenesis.

## 4 S100A8/A9 and cardiac arrhythmogenesis

Currently, there is limited data examining the involvement of S100A8/A9 in cardiac arrhythmogenesis. However, several potential pathways through which S100A8/A9 may exhibit proarrhythmic effects deserve to be discussed.

**Figure 4:**
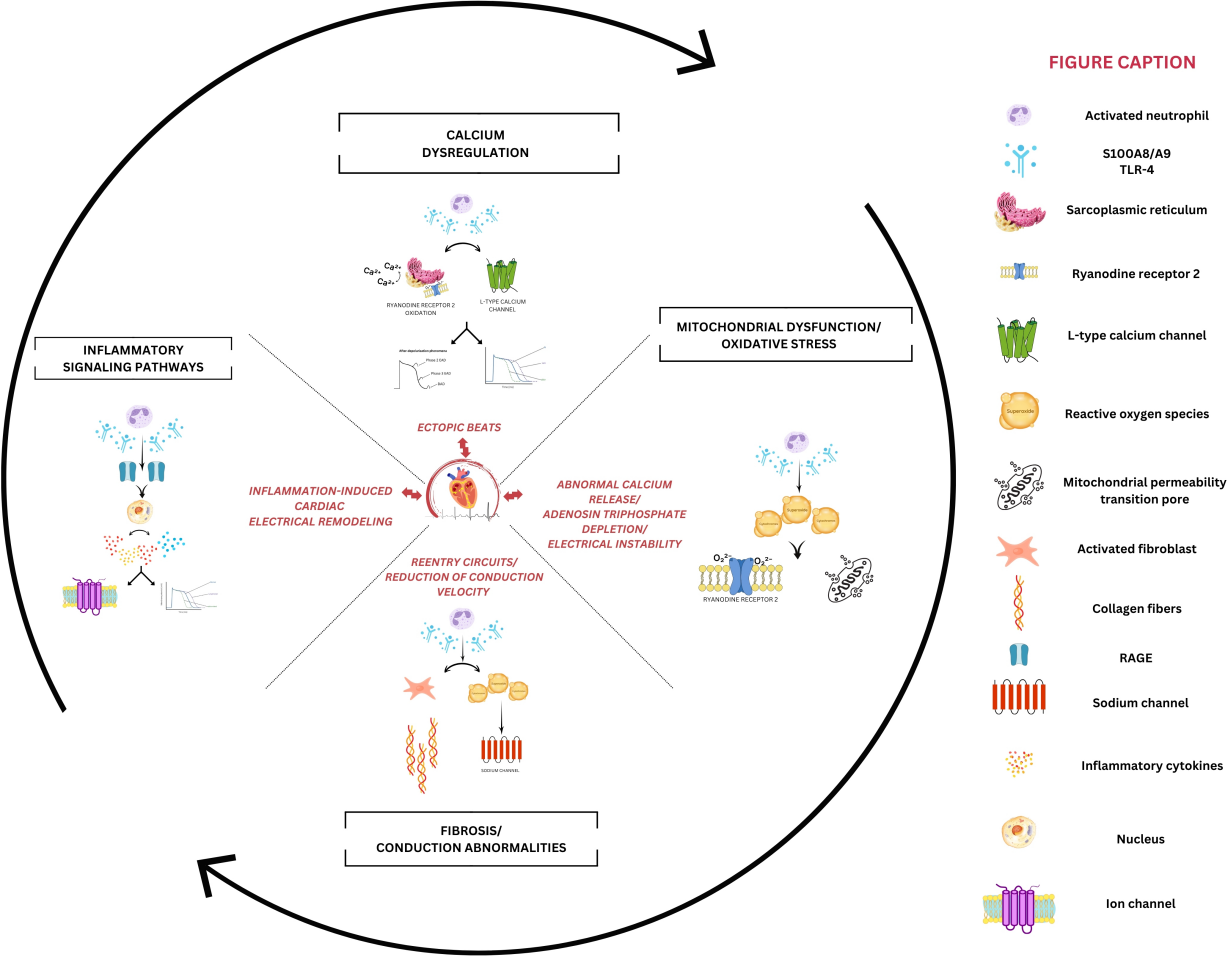
Potential mechanisms of S100A8/A9-mediated cardiac arrhythmogenesis. The left panel depicts inflammatory signaling where S100A8/A9 activates receptor for advanced glycation end-products (RAGE) and Toll-like receptors-4 (TLR4), triggering nuclear factor kappa b (NF-κB)-mediated upregulation of proinflammatory cytokines. This sustained inflammatory status indirectly alters ion channel function, disrupting normal action potential characteristics. The right panel demonstrates how S100A8/A9 induces mitochondrial dysfunction through excessive reactive oxygen species (ROS) generation. The resulting oxidative stress directly impacts the calcium-handling process, while promoting mitochondrial permeability transition pore (mPTP) opening and depleting adenosine triphosphate (ATP) reserves, which is crucial for maintaining cardiac electrical stability. The upper panel highlights calcium dysregulation, where S100A8/A9-mediated oxidation of ryanodine receptor 2 (RyR2) channels promotes sarcoplasmic reticulum calcium leak, creating a substrate for delayed after depolarizations. Concurrent L-type calcium channel dysfunction prolongs the QT interval and generates abnormal inward currents, which promotes ectopic beat formation. The lower panel emphasizes fibrotic remodeling initiated by S100A8/A9-activated fibroblasts, which will slow conduction and facilitate reentry circuits.

The three-condition framework for arrhythmia occurrence (i.e., substrate, trigger, and modulating factors) is a well-recognized concept in electrophysiology, and S100A8/A9 seems to influence all of them (**Figure 4**). In the early phase of myocardial infarction, S100A8/A9 released from neutrophils leads to the activation of proinflammatory signaling pathways, such as NF-κB or MAPK, that recruit additional inflammatory cells and promote the release of inflammatory cytokines, amplifying the inflammatory response (**Figure 4**). This initial inflammatory phase is critical for removing cellular debris; however, excessive or prolonged inflammation can lead to structural adverse cardiac remodeling, which is responsible for producing the substrate of arrhythmogenesis (87, 88).

Jakobsson et al. investigated the therapeutic potential of S100A8/A9 blockade in the context of sepsis-induced myocardial dysfunction, highlighting the critical role of S100A8/A9 in mediating both myocardial and systemic inflammation in sepsis. The study demonstrated a strong association between elevated plasma levels of S100A8/A9 and the severity of left ventricular dysfunction in patients with sepsis. Utilizing a murine model of endotoxemia, it was observed that S100A8/A9 levels increased rapidly after lipopolysaccharide administration, corresponding with the onset of cardiac dysfunction. This association suggests that S100A8/A9 may play a direct role in the inflammatory response associated with sepsis. The proinflammatory actions of S100A8/A9 appear to be mediated, at least in part, through its interactions with TLR-4 and RAGE receptors, which activate downstream inflammatory pathways. This causes the release of a range of pro-inflammatory cytokines, such as IL-1β, TNF-α, and interferon-γ, which exacerbate the inflammatory response and myocardial injury (**Figure 4**). Notably, the blocking of S100A8/A9 resulted in a significant reduction in the levels of these cytokines, both systemically and within the myocardium (69). While chronic cardiac inflammation involving S100A8/A9 via RAGE and TLR-4 is responsible for inducing arrhythmogenic myocardial substrate, this study demonstrates that acute S100A8/A9-mediated inflammation directly triggers arrhythmic events. This suggests that S100A8/A9 plays a dual role by both creating long-term structural vulnerability and interacting with immediate triggers, highlighting a more complex relationship between inflammation and cardiac electrical abnormalities than previously understood (89, 90).

Interestingly, S100A8/A9 also exhibits anti-inflammatory properties. The current literature establishes that S100A8/A9 possesses context-dependent anti-inflammatory effects that can limit post-injury cardiac inflammation and fibrosis, mechanisms highly relevant to arrhythmogenesis (91, 92). However, direct experimental evidence linking these anti-inflammatory actions of S100A8/A9 to concrete arrhythmia outcomes or electrophysiological endpoints is presently lacking. However, it can inhibit NF-κB activation, reducing the production of proinflammatory cytokines (91, 92). Furthermore, it lowers the mRNA expression of inflammatory molecules, such as IL-6, and promotes the differentiation of proinflammatory monocytes into anti-inflammatory macrophages, both of which help to resolve inflammation (93). This shift toward anti-inflammatory cells promotes tissue repair and resolution of the inflammatory response. The specific mechanisms underlying these opposing actions (pro- versus anti-inflammatory) are not fully understood and may involve different oligomeric forms of S100A8/A9 or post-translational changes (92, 94, 95). Oxidized forms of S100A8/A9 can reduce neutrophil adhesion, leukocyte-driven injury, and inflammation in the injured myocardium, blunting excessive acute inflammation that promotes fibrotic and electrical remodeling (94). Also, as previously discussed, short-term S100A8/A9 blockade reduces immune infiltration and preserves cardiac function (65). Acute inhibition of S100A8/A9 in murine MI and myocarditis models lessens macrophage/neutrophil infiltration, decreases fibrosis, improves neovascularization, and protects against decline in LV function, which is crucial for harnessing the anti-inflammatory benefit, including in S100A8/A9-derived arrhythmogenesis (65). Flevari et al. observed that low levels of S100A8/A9 were associated with an increased number of ventricular tachycardia episodes in patients with stable HF over a follow-up period of approximately 48 months, suggesting the complexity of this heterodimer in cardiac arrhythmogenesis (68).

The dual nature of S100A8/A9 complicates its role in cardiovascular disease. While it exacerbates early inflammatory responses, its anti-inflammatory actions later in the process contribute to tissue repair. This dualistic nature makes S100A8/A9 a complex biomarker, potentially reflecting disease severity or even predicting prognosis, depending on the specific context and phase of the disease. Further research is needed to understand these variations and find specific treatment strategies for targeting S100A8/A9 in CVD.

S100A8/A9 also promotes fibrosis under a variety of situations by increasing the production of pro-fibrotic factors. It can stimulate fibroblasts through several signaling pathways. S100A8/A9 interacts with TLR-4 and RAGE receptors on fibroblasts, leading to increased synthesis of transforming growth factor-β (TGF-β), a powerful pro-fibrotic cytokine. Increased TGF-β levels promote fibroblast differentiation into myofibroblasts, which increase collagen production. S100A8/A9 can potentially indirectly promote fibrosis by recruiting inflammatory cells to the affected area within the heart. These cells produce more pro-fibrotic factors, contributing to the acceleration of the fibrotic process (71, 96).

S100A8/A9-induced inflammation via TLR-4 and RAGE activation causes myocardial injury, fibrosis, immune cell infiltration, and electrophysiological alterations, all of which increase the risk of cardiac arrhythmias (87–89). These alterations disrupt the heart’s normal electrical conduction system, promote abnormal automaticity, and increase the likelihood of re-entry circuits, all of which are major triggers of arrhythmias.

The direct mechanistic link to ion channel remodeling is best supported for Ca^2+^ handling, with less evidence for other channels. The translational relevance is uncertain due to the lack of direct human mechanistic studies. The S100 protein family is involved in Ca^2+^ handling in many tissues and organs. S100A1 interacts with the ryanodine receptor (RyR2), the sarcoplasmic reticulum Ca²+-adenosine triphosphate (ATP) ase (SERCA2), and the mitochondrial F1-ATPase to modulate Ca²+ cycling within the sarcoplasmic reticulum and to influence mitochondrial function in cardiomyocytes, exhibiting antihypertrophic and antiarrhythmic effects. Particularly, the S100A1 protein appears to be significantly downregulated in individuals with severe HF, which may result in an important proarrhythmic effect in these patients (97). S100A8/A9 binding to RAGE receptors on cardiomyocytes in sepsis might lead to cardiomyocyte dysfunction observed in experimental studies due to a decrease in cardiomyocyte contractility, possibly through their interaction with the calcium-regulating proteins SERCA2 and/or RyR2, thereby altering intracellular Ca^2+^ handling (70). However, little is known about the involvement of S100A8/A9 in Ca^2+^ handling within the heart. It is expected to have both direct effects on cardiomyocytes (potentially altering Ca^2+^ influx and the function of Ca^2+^-handling proteins) and indirect effects mediated through inflammation and mitochondrial dysfunction. While direct K^+^ channel interactions with S100A8/A9 haven’t been demonstrated, related S100 proteins modulate calcium-activated K^+^ channels, background K^+^ currents through TWIK-related Acid Sensitive K^+^ channels-1, and voltage-dependent K^+^ channels (98). There are no direct studies demonstrating S100A8/A9 effects on human inward rectifier in the voltage-gated potassium channel family, while detailed electrophysiological studies with patch-clamp recordings of S100A8/A9 effects on specific ionic currents are unavailable. S100A8/A9-induced calcium handling abnormalities would secondarily affect all calcium-dependent potassium currents, altering repolarization patterns and action potential duration. Moreover, the protein’s ability to compete with calmodulin for regulatory binding sites suggests potential direct interactions with voltage-gated potassium channels that use calmodulin for regulation (98).

Direct studies examining S100A8/A9 effects on gap junctions and connexin proteins are notably absent from the current literature, representing a significant research gap. However, indirect evidence suggests meaningful effects on intercellular coupling. The structural remodeling promoted by S100A8/A9 affects gap junction distribution and function. S100A8/A9 stimulates fibroblast proliferation and collagen type III expression through RAGE signaling, creating fibrotic tissue that disrupts normal gap junction coupling patterns (71, 96). These effects can have a significant impact on heart inotropism, lusitropism, and electrical stability, leading to the development of systolic dysfunction and increased susceptibility to atrial and ventricular arrhythmias.

The complex involvement of S100A8/A9 in cardiac damage and dysfunction also includes its influence on mitochondrial function. S100A8/A9’s impact on mitochondria seems not to be a direct one, but rather a consequence of its influence on inflammation and oxidative stress within the heart. The primary mechanism by which S100A8/A9 contributes to mitochondrial dysfunction involves the disruption of the electron transport chain. Specifically, S100A8/A9, through the TLR-4 mediated signaling pathway, downregulates the expression of NDUF genes, which regulate the complex I (ubiquinone oxidoreductase subunit) in the electron transport chain of the mitochondria (47). The inhibition of complex I activity results in decreased ATP production and increased ROS formation, both of which are markers of mitochondrial dysfunction. The reduction in the peroxisome proliferator-activated receptor gamma coactivator 1-alpha (PGC-1α)//nuclear respiratory factor 1 (NRF1) signaling further exacerbates this process, as these factors are essential for mitochondrial biogenesis and function (99). Emerging evidence from recent studies indicates a significant role for PGC-1α in cardiac electrophysiology. These investigations suggest that PGC-1α contributes to the substrate that predisposes the myocardium to arrhythmogenic events. Furthermore, a potential link between PGC-1α and the regulation of Na^+^/Ca^2+^ homeostasis has been proposed, suggesting that alterations in PGC-1α expression or activity may influence the balance of intracellular calcium, a critical determinant of cardiac excitability and contractility (100, 101).

Studies on mice with S100A9 deletion have shown that S100A9 has a protective effect against sepsis-induced heart damage, as seen by reduced mitochondrial dysfunction, apoptosis, and pro-inflammatory cytokine production. In contrast, unfavorable effects are increased in transgenic mice overexpressing S100A8/A9. Pharmacological inhibition of S100A8/A9 with the small molecule inhibitor ABR-238901 showed promise in reducing mitochondrial dysfunction, suggesting that S100A8/A9 inhibition may play a role in reducing arrhythmic burden (69).

While no studies have directly linked the S100A8/A9 protein to cardiac arrhythmia onset or maintenance, its established effects on the triad inflammation, calcium handling, and mitochondrial function, all of which are interconnected in arrhythmogenesis, suggest that S100A8/A9 may play an indirect role in cardiac arrhythmogenicity.

## 5 S100A8/A9 in inflammation: a double-edged mediator

The paradoxical dual roles of S100A8/A9 represent a fundamental challenge in understanding its pathophysiology. Rather than being simply pro- or anti-inflammatory, S100A8/A9 functions as a molecular switch whose effects depend on multiple regulatory factors. In terms of concentration-dependent effects, nanomolar ranges (low concentrations) are associated with cell growth, survival signaling, and wound healing responses. At these levels, S100A8/A9 acts as a damage signal that initiates protective responses without overwhelming cellular defenses (102). Short-term pharmacologic blockade (3 days) of S100A8/A9 after myocardial infarction significantly reduces acute inflammatory cell infiltration and tissue damage, improving early and long-term cardiac function (24, 51). Prolonged or extended blockade (>7 days post-myocardial infarction) of S100A8/A9 has adverse effects, such as impaired monocyte/hematopoietic stem cell responses, reduced reparative/fibrotic macrophage recruitment, and deterioration of cardiac function (36, 51). Utilizing single-cell RNA sequencing and mass cytometry to track monocyte-derived cells following myocardial infarction, Shen et al. identified S100A9 macrophages as pivotal mediators of both acute inflammation and subsequent fibrotic remodeling. While their findings establish a direct involvement of S100A9-expressing macrophages in both the injury and remodeling phases, they do not explore the role of concentration gradients in these processes (103). As previously discussed, most data on S100 proteins’ dual role and threshold values derive from rheumatological studies, where increased concentrations of S100 proteins in serum and synovial fluid closely correlate with disease activity in several rheumatic diseases and serve as useful biomarkers for monitoring disease activity. S100A8/A9 proteins exhibit concentration-dependent functional duality, wherein dimeric forms at low-micromolar concentrations engage TLR4 to drive leukocyte adhesion, migration, and classic pro-inflammatory responses (65, 104). Conversely, tetrameric configurations achieved through elevated Ca^2+^ levels or local concentration accumulation at high-micromolar ranges (250 ng mL^−1^) lose TLR4 activity due to binding site masking, instead eliciting anti-inflammatory and regulatory effects, including suppression of monocyte dynamics, interaction with CD69, and spatial restriction of inflammatory processes (104). However, current research has not accurately established quantitative thresholds that define specific Ca^2+^ and protein concentrations at which oligomerization transitions occur in patients with ASCVD and HF.

Even though it is well-demonstrated that high concentrations of S100A8/A9 promote inflammation and lower concentrations may facilitate resolution, direct *in vivo* titration or measurement of physiologically relevant thresholds is not performed in these key cardiac studies, and most experiments employ complete pharmacologic blockade. Cellular context (macrophage/neutrophil source) is examined to a greater degree than absolute tissue levels. In neutrophils, S100A8/A9 contributes to antimicrobial activity and the formation of NETs. In monocytes and macrophages, it promotes the production of pro-inflammatory mediators, while in endothelial cells, S100A8/A9 upregulates the expression of adhesion molecules, facilitating leukocyte recruitment (16–18). Understanding this duality is essential not only for comprehending inflammatory pathophysiology but also for developing nuanced therapeutic strategies that harness protective functions while preventing pathological consequences.

The small-molecule pharmacological inhibitor ABR-238901 selectively targets S100A9 and has demonstrated efficacy in reducing infarct size and enhancing cardiac function in preclinical models of myocardial infarction. Emerging translational studies suggest that short-term administration (3–5 days) following infarction may offer an optimal balance between attenuating inflammation and preserving reparative processes (24, 36, 76). A recent study has demonstrated that early administration of ABR-238901 (30 mg/kg/day) during the first 7 days following experimentally induced myocarditis in mice significantly improved survival, enhanced cardiac function, and reduced myocardial inflammatory responses (105). Moreover, Vlad et al. demonstrated that a 3-day administration of ABR-238901 in an experimental mouse model of myocardial infarction conferred cardioprotective effects, primarily through the inhibition of S100A8/A9-induced upregulation of NADPH oxidase expression and activation of the NLRP3 inflammasome (106). These findings are consistent with previous data in myocardial infarction models and further support the concept that short-term S100A9 blockade offers a favorable therapeutic window by mitigating inflammation while preserving reparative mechanisms.

## 6 Gaps in knowledge and future directions

Despite significant advances in understanding the role of S100A8/A9 in coronary atherosclerosis, HF, and cardiac arrhythmogenesis, several critical knowledge gaps warrant further investigation, especially in terms of mechanistic understandings and clinical translation from experimental studies.

The exact molecular mechanisms by which S100A8/A9 exerts its effects in various cardiovascular pathologies remain incompletely explored. While receptor-mediated signaling through TLR-4 and RAGE has been established, the downstream intracellular cascades show tissue- and context-dependent variations that require further elucidation. Particularly unclear is how the same molecular complex can induce dual effects by promoting inflammation and tissue damage in some contexts while facilitating repair in others. Apart from that, the functions of S100A8/A9 in cardiomyocytes and their relationship with intracellular calcium homeostasis abnormalities in arrhythmogenesis represent a significant knowledge gap. The relationship between S100A8/A9 and classic regulators of cardiac electrophysiology also remains poorly characterized.

Our current understanding is limited by cross-sectional studies that provide brief snapshots, rather than longitudinal insights. The temporal dynamics of S100A8/A9 expression throughout disease progression, from subclinical atherosclerosis to plaque rupture or from compensated to decompensated HF, remain poorly described. To date, it is unknown whether S100A8/A9 serves primarily as an early mediator or a risk factor per se in cardiovascular pathology.

The translation of findings from experimental models to clinical applications represents another important gap, due to differences between murine and human S100A8/A9 biology, particularly regarding downstream signaling, which may complicate interpretation. Moreover, the biomarker potential of S100A8/A9 is limited by a lack of standardization in measurement methods and insufficient large-scale longitudinal studies correlating S100A8/A9 levels with clinically significant outcomes.

The ubiquitous expression of S100A8/A9 in inflammation imposes significant challenges for developing therapeutic approaches, since complete inhibition may compromise its beneficial functions in tissue repair. Understanding the structural details of S100A8/A9 biological interactions with various inflammatory cells could enable a targeted, selective modulation of specific pathways, while preserving S100A8/A9’s beneficial effects. Several promising future directions may address these gaps. Transcriptomics and proteomics could help to clarify the interaction of S100A8/A9 with cardiovascular pathology, while the recent development of molecular imaging techniques may allow *in vivo* monitoring of S100A8/A9 expression in atherosclerotic plaques and cardiomyocytes.

The dichotomous roles of S100A8/A9 require further insights into their time, cell, and context-dependent involvement in the pathogenesis of CVD, to allow identification of the therapeutic window and tailored therapies. Further studies should focus on establishing optimal timing for S100A8/A9 blockade intervention, investigating the transition point between pro-inflammatory and reparative phases in myocardial injury to maximize the therapeutic benefit. Developing temporal biomarker profiles to guide personalized treatment initiation and duration represents another research priority for S100A8/A9 involvement in CVD. While the role of S100A8/A9 in atherosclerosis and HF is increasingly understood, its involvement in cardiac arrhythmogenesis remains poorly understood. Therefore, future studies should investigate the impact of S100A8/A9 on the cardiac conduction system and autonomic regulation, the role in atrial fibrillation initiation, but also the contribution to ventricular arrhythmias in post-myocardial infarction patients. To bridge the gap between preclinical findings and clinical application, phase I/II trials of S100A8/A9 inhibitors in a specific CVD population are required. These research strategies offer promising pathways for advancing our understanding of S100A8/A9 in CVD and translating this knowledge into effective therapeutic strategies.

## 7 Conclusion

S100A8/A9 has emerged as a key mediator in several cardiovascular conditions, contributing to plaque destabilization and rupture in ACS, promoting arrhythmogenesis through effects on ion channel function, automatism, and structural remodeling, and playing a role in left ventricular systolic and diastolic dysfunction. These findings highlight its potential both as a biomarker and therapeutic target in ASCVD, HF, and cardiac arrhythmogenesis. However, its stage-dependent effects and mechanistic ambiguity must be addressed for clinical translation to be effective.
